# A Phase 2 Proof‐of‐Concept, Randomized, Placebo‐Controlled Trial of CX‐8998 in Essential Tremor

**DOI:** 10.1002/mds.28584

**Published:** 2021-03-25

**Authors:** Spyros Papapetropoulos, Margaret S. Lee, Stacey Versavel, Evan Newbold, Hyder A. Jinnah, Rajesh Pahwa, Kelly E. Lyons, Rodger Elble, William Ondo, Theresa Zesiewicz, Peter Hedera, Adrian Handforth, Jenna Elder, Mark Versavel

**Affiliations:** ^1^ Massachusetts General Hospital Boston Massachusetts USA; ^2^ Jazz Pharmaceuticals Philadelphia Pennsylvania USA; ^3^ Cerevel Therapeutics, LLC Boston Massachusetts USA; ^4^ Emory University School of Medicine Atlanta Georgia USA; ^5^ University of Kansas Medical Center Kansas City Kansas USA; ^6^ Southern Illinois University School of Medicine Springfield Illinois USA; ^7^ Houston Methodist Neurological Institute Houston Texas USA; ^8^ University of South Florida Ataxia Research Center Tampa Florida USA; ^9^ Department of Neurology University of Louisville Louisville Kentucky USA; ^10^ VA Greater Los Angeles Healthcare System Los Angeles California USA; ^11^ PharPoint Research, Inc. Wilmington North Carolina USA; ^12^ vZenium, LLC Arlington Massachusetts USA

**Keywords:** CX‐8998, T‐Type calcium channel modulator, essential tremor, T‐CALM, phase 2 trial

## Abstract

**Background:**

Available essential tremor (ET) therapies have limitations.

**Objectives:**

The objective of this study was to evaluate CX‐8998, a selective T‐type calcium channel modulator, in essential tremor.

**Methods:**

Patients 18–75 years old with moderate to severe essential tremor were randomized 1:1 to receive CX‐8998 (titrated to 10 mg twice daily) or placebo. The primary end point was change from baseline to day 28 in The Essential Tremor Rating Assessment Scale performance subscale scored by independent blinded video raters. Secondary outcomes included in‐person blinded investigator rating of The Essential Tremor Rating Assessment Scale performance subscale, The Essential Tremor Rating Assessment Scale activities of daily living subscale, and Kinesia ONE accelerometry.

**Results:**

The video‐rated The Essential Tremor Rating Assessment Scale performance subscale was not different for CX‐8998 (n = 39) versus placebo (n = 44; *P* = 0.696). CX‐8998 improved investigator‐rated The Essential Tremor Rating Assessment Scale performance subscale (*P* = 0.017) and The Essential Tremor Rating Assessment Scale activities of daily living (*P* = 0.049) but not Kinesia ONE (*P* = 0.421). Adverse events with CX‐8998 included dizziness (21%), headache (8%), euphoric mood (6%), and insomnia (6%).

**Conclusions:**

The primary efficacy end point was not met; however, CX‐8998 improved some assessments of essential tremor, supporting further clinical investigation. © 2021 The Authors. *Movement Disorders* published by Wiley Periodicals LLC on behalf of International Parkinson and Movement Disorder Society. This article has been contributed to by US Government employees and their work is in the public domain in the USA.

Essential tremor (ET) is a common, progressive movement disorder that profoundly affects activities of daily living (ADLs).^1^, ^2^, ^3^, ^4^, ^5^, ^6^ As there is no cure, treatment is symptomatic.^5^ Only 30%–70% of patients report some improvements with first‐line treatments (propranolol and primidone).^6^

Although the pathogenesis of ET has not been fully established, abnormal oscillations of neuronal activity in the cortico‐bulbo‐cerebello‐thalamic pathways are believed to be involved.^5^ Increased activation of T‐type calcium channels promotes excessive rhythmicity in these neural networks.^7^, ^8^, ^9^, ^10^, ^11^

CX‐8998 is a T‐type calcium channel modulator with low nanomolar potency against all 3 isoforms and >100‐fold selectivity compared with other ion channels.^12^ This proof‐of‐concept study evaluated the efficacy, safety, and tolerability of CX‐8998 in patients with moderate to severe ET.

## 1 Methods

The design of T‐CALM (ClinicalTrials.gov: NCT03101241) was previously described^13^ and is briefly summarized here. Ethical conduct was consistent with regulatory guidelines.

### 1.1 Study Design and Participants

T‐CALM was a phase 2 multicenter double‐blind, randomized, placebo‐controlled trial of CX‐8998, titrated to a target dosage of 10 mg twice daily (20 mg/day), for a total of 28 days, in patients with moderate to severe ET. Patients were 18–75 years of age and diagnosed with classic bilateral ET^14^ before age 65. Eligible participants had tremor severity score of ≥2 in ≥1 arm during any of the 3 maneuvers of The Essential Tremor Rating Assessment Scale performance subscale (TETRAS‐PS) item 4 (maneuver 1, upper limbs held forward and horizontally; 2, upper limbs extended laterally and horizontally with elbows flexed, and hands positioned close to each other near chin; 3, finger‐nose or finger‐chin movements) and TETRAS‐PS total score ≥ 15 at screening. Use of a stable dosage of a single antitremor medication (with the exception of primidone) was permitted during the study.

### 1.2 Procedures

Patients were randomized 1:1 to receive CX‐8998 or placebo, stratified by concomitant use of antitremor medication and study site.

Patients received titrated dosages of CX‐8998 during a 4‐week double‐blind dosing period (4, 8, and 10 mg twice daily during weeks 1, 2, and 3–4, respectively) and were evaluated for safety and efficacy on days 15 (beginning in week 3) and 28 (end of week 4). The dose of study medication could be reduced to the next lower level, if needed; only 1 dose reduction was allowed. Target dose and titration schedule were based on the tolerability profile of CX‐8998 (immediate‐release formulation) in a prior clinical study.^15^

### 1.3 Outcomes

The Essential Tremor Rating Assessment Scale (TETRAS) comprises a 9‐item performance subscale (TETRAS‐PS) and a 12‐item ADL subscale (TETRAS‐ADL).^16^ Each patient's TETRAS‐PS assessment was scored by investigators in real time at select study visits and by 1 of 3 independent raters of video recordings made during the on‐site ratings. Both sets of scores were analyzed using identical methodology.

The primary end point was TETRAS‐PS change from baseline to day 28 scored by independent video raters. TETRAS‐PS change from baseline to day 28 was also scored in person by investigators. Secondary end points included TETRAS‐ADL change from baseline to day 28, rated by patients during an investigator‐led interview, and accelerometry with Kinesia ONE.^17^, ^18^ Exploratory end points included change from baseline to day 15 and day 28 in TETRAS total score (investigator‐rated TETRAS‐PS plus TETRAS‐ADL); change from baseline to day 15 in TETRAS‐PS, TETRAS‐ADL, and Kinesia ONE; and ratings of improvement on day 15 and day 28 measured by Clinical Global Impression of Improvement (CGI‐I) and Patient Global Impression of Change (PGIC).

Safety assessments included treatment‐emergent adverse events (TEAEs), physical examination, neurologic examination, vital signs, clinical laboratory tests, electrocardiogram, and Columbia Suicide Severity Rating Scale.

### 1.4 Statistical Analysis

Approximately 92 patients were planned for enrollment to ensure 86 patients for efficacy analyses. A sample size of 43 patients per treatment group had ≥90% power to detect at least a 5.5‐point difference between CX‐8998 and placebo for the primary end point, assuming a standard deviation (SD) of 7.5 and α = 0.05.^19^ This calculation was based on the Wilcoxon‐Mann–Whitney test for 2 independent means and assumed normal distributions for each treatment group with a common, but unconfirmed, SD.

The primary efficacy end point was analyzed with an analysis of covariance model, with fixed effects for treatment, antitremor medication use, study site, and baseline TETRAS‐PS score. Secondary and exploratory end points were analyzed similarly.

The intention‐to‐treat (ITT) analysis set included all randomized subjects. The full analysis set, used for efficacy assessments, included all patients who received any study drug and had both a baseline assessment and ≥1 postbaseline efficacy assessment. The safety analysis set included ITT patients who received any study drug.

## 2 Results

### 2.1 Patients

The ITT population included 95 patients (CX‐8998, n = 48; placebo, n = 47); the full analysis set comprised 83 patients (CX‐8998, n = 39; placebo, n = 44; Fig. S1). The maximum dose of study drug was reached by 38 of 48 patients (79%) on drug and 42 of 47 patients (89%) on placebo.

About half the population (47%) was female. Mean ± SD age was 63 ± 10.2 years. Mean ± SD time since onset of ET was 23 ± 16.0 years (Table 1). The treatment groups were matched for most baseline characteristics. Compliance with study drug administration was comparable for CX‐8998 (99.3%) and placebo (97.7%).

**TABLE 1:** Demographic and essential tremor characteristics at baseline (ITT population)

Parameter	CX‐8998 (n = 48)	Placebo (n = 47)	Total (n = 95)
Sex			
Male	25 (52%)	25 (53%)	50 (53%)
Female	23 (48%)	22 (47%)	45 (47%)
Age at informed consent, y			
Mean (SD)	64 (9.6)	63 (10.8)	63 (10.2)
Median	66	66	66
Minimum, maximum	28, 75	21, 75	21, 75
Age group			
≤65 y	22 (46%)	22 (47%)	44 (46%)
>65 y	26 (54%)	25 (53%)	51 (54%)
Race			
White	45 (94%)	46 (98%)	91 (96%)
Black or African American	3 (6%)	1 (2%)	4 (4%)
Ethnicity			
Not Hispanic or Latino	48 (100%)	45 (96%)	93 (98%)
Hispanic or Latino	0 (0%)	1 (2%)	1 (1%)
Not reported	0 (0%)	1 (2%)	1 (1%)
Time since onset of essential tremor, y^a^			
Mean (SD)	24 (16.3)	21 (15.7)	23 (16.0)
Median	20	18	19
Minimum, maximum	3, 63	1, 62	1, 63
Essential tremor improves with alcohol			
Yes	23 (48%)	16 (34%)	39 (41%)
No	11 (23%)	13 (28%)	24 (25%)
Unknown	14 (29%)	18 (38%)	32 (34%)
Baseline TETRAS‐PS total score (independent video rated)^b^			
Mean (SD)	23.1 (6.3)	22.8 (5.7)	22.9 (6.0)
Median	22.3	22.5	22.5
Minimum, maximum	11.5, 46.5	12.5, 43.5	11.5, 46.5
Baseline TETRAS‐PS total score (investigator‐rated)^b^			
Mean (SD)	28.4 (5.9)	28.6 (6.4)	28.5 (6.1)
Median	27.3	27.5	27.5
Minimum, maximum	20.0, 42.5	19.5, 45.0	19.5, 45.0
Baseline TETRAS‐ADL subscale score^b^			
Mean (SD)	26 (6.0)	26 (7.0)	26 (6.5)
Median	26	26	26
Minimum, maximum	13, 38	9, 42	9, 42
Baseline TETRAS total score^b^ ^,^ ^c^			
Mean (SD)	49.2 (10.6)	48.9 (10.5)	49.1 (10.5)
Median	47.0	48.5	47.5
Minimum, maximum	28.0, 81.5	28.5, 85.5	28.0, 85.5
Using primidone at screening^d^	5 (10%)	6 (13%)	11 (12%)
Antitremor medication at study entry^e^	22 (46%)	21 (45%)	43 (45%)

ADL, activities of daily living; ITT, intention to treat; SD, standard deviation; TETRAS, The Essential Tremor Rating Assessment Scale; TETRAS‐PS, TETRAS performance subscale (independent video rated unless otherwise noted).

^a^ Time since onset of essential tremor was estimated by subtracting age at onset of essential tremor from age at informed consent.

^b^ Baseline was defined as the last nonmissing value that was obtained before or ≤15 minutes after initiation of study drug.

^c^ TETRAS total score is sum of TETRAS‐PS subscale total score (independent video rated) and TETRAS‐ADL subscale score.

^d^ Use of primidone at screening was permitted; however, the screening period was to be extended by 2 weeks, for a total of 6 weeks, and primidone was to be discontinued.

^e^ Screening until baseline.

### 2.2 Efficacy

**FIG. 1:**
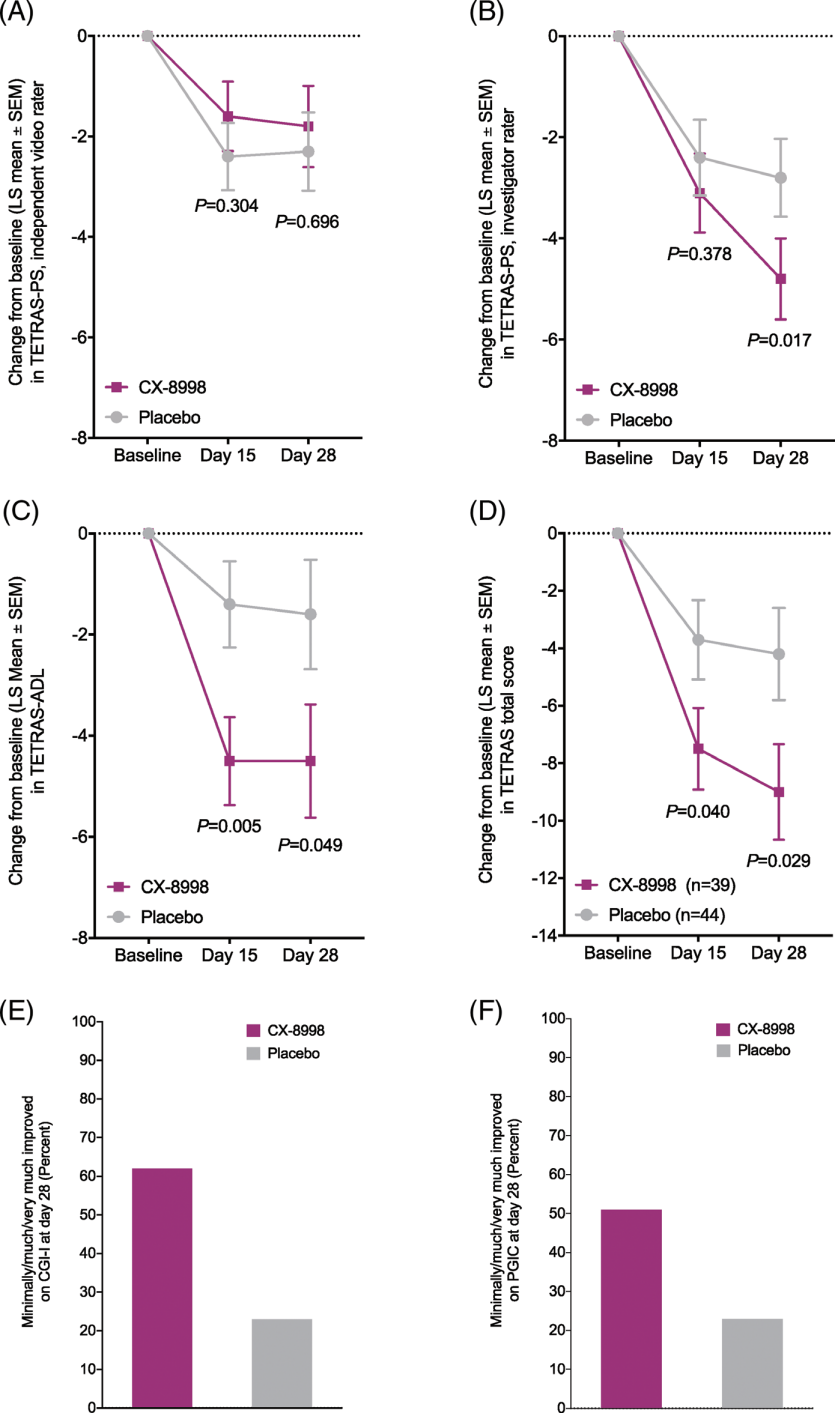
(**A**) Change from baseline to days 15 and 28 in TETRAS‐PS, as scored by independent video raters (primary efficacy end point). (**B**) Change from baseline to days 15 and 28 in TETRAS‐PS, as scored by investigators. (**C**) Change from baseline to days 15 and 28 in TETRAS‐ADL. (**D**) Change from baseline to days 15 and 28 in TETRAS total score. (**E**) Percentage of patients rated as minimally/much/very much improved on the CGI‐I on day 28. (**F**) Percentage of patients rated as minimally/much/very much improved on the PGIC on day 28. To facilitate comparisons, the CGI‐I and PGIC responses were mapped to values from −3 to 3 (from worst outcome to best outcome). The full analysis set was used in all analyses. *P* values shown in B–D are nominal. For more details, see Tables [Link], [Link], and S4. CGI‐I, Clinical Global Impression of Improvement; LS, least squares; PGIC, Patient Global Impression of Change; TETRAS, The Essential Tremor Rating Assessment Scale; TETRAS‐PS, The Essential Tremor Rating Assessment Scale performance subscale; TETRAS‐ADL, The Essential Tremor Rating Assessment Scale activities of daily living subscale.

The difference between treatment groups on change from baseline to day 28 in TETRAS‐PS assessments scored by independent video raters (primary end point) was not statistically significant (least‐squares [LS] mean ± SE, −1.8 ± 0.81 for CX‐8998 vs −2.3 ± 0.78 for placebo; *P* = 0.696; Fig. 1A). In contrast, TETRAS‐PS assessments rated by investigators in person showed improvements with CX‐8998 versus placebo on day 28 (LS mean ± SE changes from baseline, −4.8 ± 0.80 for CX‐8998 vs −2.8 ± 0.77 for placebo; *P* = 0.017; Fig. 1B). Intraclass correlation coefficient (ICC) was calculated from a subset of data in which 4 videos were each scored by 4 independent video raters and 1 investigator. The ICC among the video raters was 0.80. When investigator ratings were included in the analysis, the ICC was reduced to 0.60; although this was a limited data analysis, this may be reflective of the discrepancies between video and investigator raters.

Compared with placebo, CX‐8998 improved TETRAS‐ADL scores on day 15 (LS mean ± SE changes from baseline, −4.5 ± 0.87 for CX‐8998 vs −1.4 ± 0.85 for placebo; *P* = 0.005) and day 28 (−4.5 ± 1.12 for CX‐8998 vs −1.6 ± 1.06 for placebo; *P* = 0.049; Fig. 1C). TETRAS total scores were improved with CX‐8998 versus placebo on day 15 (LS mean ± SE, −7.5 ± 1.42 for CX‐8998 vs −3.7 ± 1.38 for placebo; *P* = 0.040) and day 28 (−9.0 ± 1.66 for CX‐8998 vs −4.2 ± 1.60 for placebo; *P* = 0.029; Fig. 1D).

CX‐8998 demonstrated improvement versus placebo on the CGI‐I on day 28 (LS mean ± SE, 1.0 ± 0.13 vs 0.4 ± 0.13; LS mean difference, 0.6; 95% CI, 0.3–0.9; *P* = 0.001) and PGIC on day 15 (LS mean ± SE, 0.9 ± 0.17 vs 0.3 ± 0.16; LS mean difference, 0.7; 95% CI, 0.2–1.1; *P* = 0.003). On day 28, more patients treated with CX‐8998 were rated minimally/much/very much improved on the CGI‐I and PGIC, compared with placebo (Fig. 1E,F; Table S1).

Kinesia ONE scores (triaxial accelerometry and gyroscopy) were similar with CX‐8998 and placebo on days 15 and 28 (Table S2).

### 2.3 Safety

At least 1 TEAE was present in 58% and 49% of patients with CX‐8998 and placebo, respectively (Table S3). TEAEs were mostly mild or moderate. TEAEs with CX‐8998 were primarily neurologic and psychiatric and included dizziness (21%), headache (8%), euphoric mood (6%), and insomnia (6%). TEAEs with CX‐8998 were mostly reported during week 1 (40%, 21%, 17%, and 4% during weeks 1, 2, 3, and 4, respectively), whereas TEAEs with placebo were reported throughout the study (19%, 15%, 19%, and 9% during weeks 1, 2, 3, and 4, respectively; Table S3). TEAEs leading to discontinuation of study drug and dosage reduction are reported in Tables S4 and S5, respectively. Clinically meaningful differences were not detected in clinical laboratory parameters, vital signs, electrocardiogram, or neurologic and physical examinations.

## 3 Discussion

T‐CALM was a proof‐of‐concept study evaluating CX‐8998 in patients with moderate to severe ET. The primary efficacy end point, change from baseline to day 28 on TETRAS‐PS scored by independent video raters, was not met. However, CX‐8998 improved symptoms of ET on a closely related objective measure (investigator‐rated TETRAS‐PS), as well as TETRAS‐ADL, TETRAS total scores, and other patient‐reported and clinician‐reported end points.

The dual approach to TETRAS‐PS scoring aimed to identify the optimal rating methodology for late‐stage clinical development.^20^ The independent video rater assessment was selected for the primary end point analysis, as it was hypothesized to mitigate investigator bias and variability. The traditional methodology used scores from site investigators who observed patients in real time with the advantage of 3‐dimensional angle and depth perception. Compared with the site investigators, the independent video raters consistently scored all but 1 TETRAS‐PS subitem lower (data not shown), particularly subitems associated with limitations in videography (face, voice, lower limb, and trunk).^16^ In the CX‐8998 group but not the placebo group, this effect translated into smaller change scores with video raters versus on‐site investigators. These findings may have implications for future clinical trials. For example, adjustments may improve the videographic process for assessing tremor severity, as size perception is altered (items are perceived as smaller) on images viewed through small 2‐dimensional computer screens.^21^ In addition, in‐person assessments of tremor severity potentially should be performed by raters lacking knowledge of the patient's experience, as investigators may be biased by functional unblinding through other patient observations (eg, AEs, TETRAS‐ADL, PGIC).

ET has a major debilitating effect on ADLs.^1^, ^2^, ^3^, ^4^, ^22^, ^23^, ^24^, ^25^ Up to 75% of patients with ET experience impairment in ADLs such as eating, drinking, and handwriting.^26^ CX‐8998 was associated with improvement on TETRAS‐ADL, a patient‐reported outcome focusing on the functional implications of tremor for ADLs.^16^ These results support the use of TETRAS‐ADL as a primary end point in future clinical trials.

The Kinesia ONE device, which produces algorithmically derived scores for postural and kinetic tremor in the upper limbs,^17^, ^18^ was explored as a potential digital biomarker of tremor severity. However, Kinesia ONE scores were not impacted by CX‐8998. This could be related to technical problems (eg, variable finger sensor placement). Future studies with this device and digital biomarkers are essential for further validation of their use in ET clinical trials.^26^

The most common TEAEs with CX‐8998 were dizziness, headache, euphoric mood, and insomnia and were mild to moderate. Compared with placebo, more patients receiving CX‐8998 withdrew because of AEs; no AE leading to discontinuation occurred in >2 patients.

In summary, CX‐8998 titrated to 10 mg twice daily ameliorated ET symptoms, although the primary end point was not met. This proof‐of‐concept study supports further clinical investigation of CX‐8998 for the treatment of ET.

## Financial Disclosures of All Authors (for the Preceding 12 Months)

S. Papapetropoulos is currently affiliated with Massachusetts General Hospital and is a former employee and shareholder of Cavion, Inc., a wholly owned subsidiary of Jazz Pharmaceuticals, a former employee and shareholder of Acadia Pharmaceuticals Inc., a current employee and shareholder of Vigil Neurosciences Inc., and owner of Encephalos Life Sciences LLC.

M.S. Lee is an employee and shareholder of Jazz Pharmaceuticals and a former employee and shareholder of Cavion, Inc., a wholly owned subsidiary of Jazz Pharmaceuticals.

S. Versavel is an employee of Cerevel Therapeutics, LLC, and a former employee and shareholder of Cavion, Inc., a wholly owned subsidiary of Jazz Pharmaceuticals.

E.J. Newbold is a former employee and shareholder of Jazz Pharmaceuticals and of Cavion, Inc., a wholly owned subsidiary of Jazz Pharmaceuticals.

H.A. Jinnah has received grant support from the National Institutes of Health, Cure Dystonia Now, Cavion, Inc. (a wholly owned subsidiary of Jazz Pharmaceuticals), and Revance Therapeutics, Inc.; has served on advisory boards or as a consultant for Allergan Inc., CoA Therapeutics, Cavion, Inc., Ipsen, and Retrophin Inc.; has received honoraria or stipends for lectures or administrative work from the American Academy of Neurology, the American Neurological Association, the Dystonia Medical Research Foundation, the International Neurotoxin Society, and the International Parkinson's Disease and Movement Disorders Society; serves on the Scientific Advisory Boards for the Benign Essential Blepharospasm Research Foundation, Cure Dystonia Now, the Dystonia Medical Research Foundation, the Tourette Association of America, and Tyler's Hope for a Cure; and is principle investigator for the Dystonia Coalition.

R. Pahwa has served as a consultant for Abbott, AbbVie, Acadia, Acorda, Adamas, Amneal, CalaHealth, DisperSol Technologies, Global Kinetics, Impel, Neuropharma, Kyowa, Lundbeck, Mitsubishi, Neurocrine, Orbis Bioscience, PhotoPharmics, Prilenia, Sunovion, Teva Neuroscience, and US World Meds; and has received research support from Abbott, AbbVie, Addex, Biogen, Biohaven, Boston Scientific, EIP, Global Kinetics, Impax, Intec, Lilly, Neuroderm, Neuraly, Parkinson's Foundation, Pharma 2B, Prelinia, Roche, Sage, SIS, Sun Pharma, Sunovion, Theranexus, Theravance, US WorldMeds, and Voyager.

K.E. Lyons is the president of the International Essential Tremor Foundation and has served as a consultant for Acorda and Abbott.

R. Elble is an employee of SIU HealthCare; has served as a consultant for Applied Therapeutics, Cadent, Cydan, Jazz, Neurocrine Biosciences, Novartis, Osmotica, Praxis Precision Medicines, and Sage; has served on advisory boards for the International Essential Tremor Foundation and the Neuroscience Research Foundation of Kiwanis International, Illinois‐Eastern Iowa District; and has received grants from the Neuroscience Research Foundation of Kiwanis International, Illinois‐Eastern Iowa District.

W. Ondo has served as a consultant for Acadia, Acorda, ADAMAS, Cadent, Merz, Neurocrine, Sage, Teva, and USWorldMeds; and has received grant support from AbbVie, Amneal, Biogen, Dystonia Coalition, Lundbeck, Restless Legs Syndrome Foundation, Revance, Sun, and Sunovion.

T. Zesiewicz has served as a consultant for Steminent Biotherapeutics; has served on advisory boards for Boston Scientific, Reata Pharmaceuticals, Inc., and Steminent Biotherapeutics; has received grant support from AbbVie Inc., Biogen, Biohaven Pharmaceutics, Boston Scientific, Bukwang Pharmaceuticals Co, Ltd, Cala Health, Inc., Cavion, Friedreich's Ataxia Research Alliance, Houston Methodist Research Institute, National Institutes of Health, Retrotope Inc., and Takeda Development Center Americas, Inc.; has served as a senior editor for *Neurodegenerative Disease Management*; and is coinventor of varenicline for treating imbalance and nonataxic imbalance.

P. Hedera has served as a consultant for Alexion Pharmaceuticals.

A. Handforth is employed by Veterans Affairs and has received grant support from Eisai.

J. Elder is an employee of PharPoint Research.

M. Versavel is an employee of vZenium, LLC, a former consultant for Cavion, Inc., a wholly owned subsidiary of Jazz Pharmaceuticals, and a current consultant for Jazz Pharmaceuticals.

## Authors’ Roles

Dr. Papapetropoulos designed and conceptualized the study, interpreted data, and revised the manuscript. Dr. Lee designed the study, interpreted data, and revised the manuscript. Dr. S. Versavel designed the study, interpreted data, directed clinical operations, and revised the manuscript. Mr. Newbold designed the study, interpreted data, and revised the manuscript. Dr. Jinnah and Dr. Pahwa were investigators and revised the manuscript. Dr. Lyons was an investigator and revised the manuscript. Dr. Elble and Dr. Ondo were independent video raters and TETRAS trainers and revised the manuscript. Dr. Zesiewicz and Dr. Hedera were investigators and revised the manuscript. Dr. Handforth revised the manuscript. Dr. Elder performed statistical analysis of data and revised the manuscript. Dr. M. Versavel designed the study and interpreted data.

## Supporting information

**Figure S1.** Study participant flow diagram. AE, adverse event; FAS, full analysis set; ITT, intention to,treat. Full analysis set: all subjects who received ≥1 dose of study drug and had both baseline and ≥1 postbaseline efficacy assessment of the same parameter for any efficacy parameter. Intention‐to‐treat set: all randomized subjects; used to display subject disposition and demographic information.

**Table S1**. CGI‐I and PGIC

**Table S2**. Kinesia ONE score

**Table S3**. Summary of treatment‐emergent AEs reported in 2 or more patients in either treatment group (safety analysis set)

**Table S4**. TEAEs leading to discontinuation of study drug

**Table S5**. TEAEs leading to dosage reductions
