# Supplementary figures and images for: A Phase 2 Proof‐of‐Concept, Randomized, Placebo‐Controlled Trial of CX‐8998 in Essential Tremor
Source: Mov Disord. 2021 Mar 25;36(8):1944–9. doi: 10.1002/mds.28584 (PMC8451783; doi:10.1002/mds.28584)

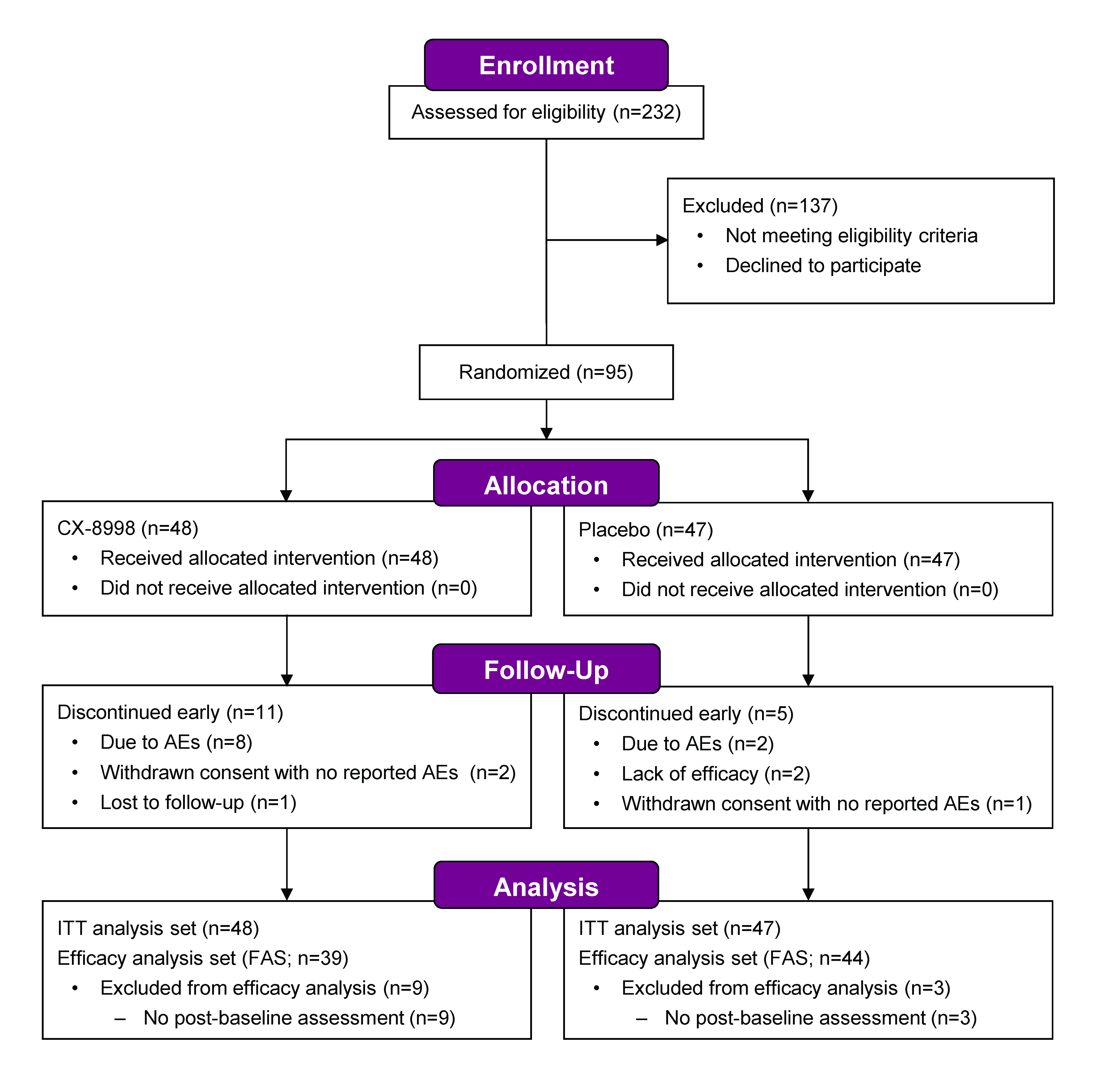

Supplement: Supplementary file 1 — Figure S1. Study participant flow diagram. AE, adverse event; FAS, full analysis set; ITT, intention to,treat. Full analysis set: all subjects who received ≥1 dose of study drug and had both baseline and ≥1 postbaseline efficacy assessment of the same parameter for any efficacy parameter. Intention‐to‐treat set: all randomized subjects; used to display subject disposition and demographic information. [file MDS-36-1944-s001.tiff]
