# Supplementary material for: A Phase 2 Proof‐of‐Concept, Randomized, Placebo‐Controlled Trial of CX‐8998 in Essential Tremor
Source: Mov Disord. 2021 Mar 25;36(8):1944–9. doi: 10.1002/mds.28584 (PMC8451783; doi:10.1002/mds.28584)
Supplement: Supplementary file 2 — Table S1. CGI‐I and PGIC [file MDS-36-1944-s006.docx]

**Supplemental Table e-1.** CGI-I and PGIC

| **Category** | | **Day 15 (Exploratory Endpoint)** | | | | **Day 28 (Exploratory Endpoint)** | | | |  |
| --- | --- | --- | --- | --- | --- | --- | --- | --- | --- | --- |
|  |  | **CX-8998 (n=39)** | | **Placebo (n=44)** | | **CX-8998 (n=39)** | | **Placebo (n=44)** | |  |
| **CGI-I^a^** | | | | | | | | | |  |
| -3 | Very much worse | 0 | | 0 | | 0 | | 0 | |  |
| -2 | Much worse | 0 | | 1 (2%) | | 0 | | 0 | |  |
| -1 | Minimally worse | 0 | | 2 (5%) | | 0 | | 2 (5%) | |  |
| 0 | No change | 14 (36%) | | 21 (48%) | | 13 (33%) | | 30 (68%) | |  |
| 1 | Minimally improved | 19 (49%) | | 14 (32%) | | 15 (38%) | | 7 (16%) | |  |
| 2 | Much improved | 6 (15%) | | 5 (11%) | | 9 (23%) | | 2 (5%) | |  |
| 3 | Very much improved | 0 | | 1 (2%) | | 0 | | 1 (2%) | |  |
|  | Minimally, much, or very much improved | 25 (64%) | | 20 (45%) | | 24 (62%) | | 10 (23%) | |  |
| **PGIC^b^** | | | | | | | | | |  |
| -3 | Very much worse | | 0 | | 0 | | 1 (3%) | | 0 | |
| -2 | Much worse | | 1 (3%) | | 4 (9%) | | 1 (3%) | | 1 (2%) | |
| -1 | Minimally worse | | 1 (3%) | | 1 (2%) | | 1 (3%) | | 2 (5%) | |
| 0 | No change | | 11 (28%) | | 27 (61%) | | 14 (36%) | | 29 (66%) | |
| 1 | Minimally improved | | 17 (44%) | | 8 (18%) | | 10 (26%) | | 6 (14%) | |
| 2 | Much improved | | 8 (21%) | | 3 (7%) | | 7 (18%) | | 2 (5%) | |
| 3 | Very much improved | | 1 (3%) | | 1 (2%) | | 3 (8%) | | 2 (5%) | |
|  | Minimally, much, or very much improved | | 26 (67%) | | 12 (27%) | | 20 (51%) | | 10 (23%) | |

^a^The CGI-I was rescaled to allow a more intuitive interpretation of the results.

^b^The PGIC was rescaled to allow a more intuitive interpretation of the results.
