# Supplementary material for: A Phase 2 Proof‐of‐Concept, Randomized, Placebo‐Controlled Trial of CX‐8998 in Essential Tremor
Source: Mov Disord. 2021 Mar 25;36(8):1944–9. doi: 10.1002/mds.28584 (PMC8451783; doi:10.1002/mds.28584)
Supplement: Supplementary file 3 — Table S2. Kinesia ONE score [file MDS-36-1944-s002.docx]

**Supplemental Table e-2.** Kinesia ONE score

| **Kinesia ONE Total Score^a^** | **Day 15** | | **Day 28** | |
| --- | --- | --- | --- | --- |
|  | **CX‑8998 (n=39)** | **Placebo (n=44)** | **CX‑8998 (n=39)** | **Placebo (n=44)** |
| Baseline, n | 39 | 44 | 39 | 44 |
| Mean (SD) | 10.6 (4.3) | 12.0 (5.4) | 10.6 (4.3) | 12.0 (5.4) |
| Median | 10.0 | 11.0 | 10.0 | 11.0 |
| Minimum, maximum | 4.3, 25.9 | 3.7, 25.0 | 4.3, 25.9 | 3.7, 25.0 |
| Day 15 or Day 28, n | 38 | 42 | 37 | 41 |
| Mean (SD) | 9.1 (4.5) | 10.8 (5.2) | 9.3 (4.3) | 10.4 (4.8) |
| Median | 7.9 | 10.1 | 7.8 | 10.3 |
| Minimum, maximum | 2.6, 27.3 | 2.8, 25.9 | 3.8, 26.0 | 3.3, 24.2 |
| Change from baseline to Day 15 or Day 28, n | 38 | 42 | 37 | 41 |
| Mean (SD) | -1.5 (3.1) | -1.4 (3.0) | -1.4 (3.4) | -1.8 (3.0) |
| Median | -1.1 | -0.8 | -0.7 | -1.3 |
| Minimum, maximum | -12.2, 4.9 | -13.9, 2.3 | -11.7, 5.4 | -13.7, 3.6 |
| Analysis results^b,c^ |  |  |  |  |
| LS mean (SE) | -2.0 (0.5) | -1.6 (0.5) | -1.7 (0.5) | -1.6 (0.5) |
| LS mean difference (95% CI) | -0.4 (-1.7, 0.9) | | 0.0 (-1.4, 1.3) | |
| p-value^c^ | 0.350 | | 0.421 | |

ANCOVA = analysis of covariance; CI = confidence interval; LS = least squares.

^a^Kinesia ONE scores are presented as the sum of the scores of the left and right hands. Values for each test range from 0 (no tremor) to 4 (severe tremor). The total overall score (32) is the sum of all individual items.

^b^LS means, standard errors, difference from placebo, 95% CIs, and p-values were estimated using an ANCOVA model, with effects for treatment, anti-tremor medication use, site type, and baseline value of the accelerometry total score.

^c^The analysis was performed on ranked data; LS means, standard errors, the difference from placebo, and 95% CIs were estimated using unranked data, and the p-value was calculated using ranked data.
