# Supplementary material for: A Phase 2 Proof‐of‐Concept, Randomized, Placebo‐Controlled Trial of CX‐8998 in Essential Tremor
Source: Mov Disord. 2021 Mar 25;36(8):1944–9. doi: 10.1002/mds.28584 (PMC8451783; doi:10.1002/mds.28584)
Supplement: Supplementary file 4 — Table S3. Summary of treatment‐emergent AEs reported in 2 or more patients in either treatment group (safety analysis set) [file MDS-36-1944-s005.docx]

**Supplemental Table e-3.** Summary of treatment-emergent AEs reported in two or more patients in either treatment group (safety analysis set)

| **MedDRA System Organ Class/ Preferred Term^a^** | **CX-8998 (n=48), n (%)** | | | | | | **Placebo (n=47), n (%)** | | | | | |
| --- | --- | --- | --- | --- | --- | --- | --- | --- | --- | --- | --- | --- |
|  | **Study Week** | | | | | | **Study Week** | | | | | |
|  | **All** | **1** | **2** | **3** | **4** | **>4** | **All** | **1** | **2** | **3** | **4** | **>4** |
| **≥1 TEAE^b^** | **28 (58)** | **19 (40)** | **10 (21)** | **8 (17)** | **2 (4)** | **1 (2)** | **23 (49)** | **9 (19)** | **7 (15)** | **9 (19)** | **4 (9)** | **1 (2)** |
| **Nervous system disorders** | **20 (42)** | **16 (33)** | **4 (8)** | **1 (2)** | **0** | **1 (2)** | **10 (21)** | **2 (4)** | **3 (6)** | **5 (11)** | **1 (2)** | **1 (2)** |
| Dizziness | 10 (21) | 9 (19) | 3 (6) | 0 | 0 | 0 | 3 (6) | 1 (2) | 0 | 2 (4) | 0 | 0 |
| Headache | 4 (8) | 4 (8) | 0 | 0 | 0 | 0 | 2 (4) | 0 | 2 (4) | 0 | 0 | 0 |
| Disturbance in attention | 2 (4) | 2 (4) | 0 | 0 | 0 | 0 | 1 (2) | 0 | 1 (2) | 0 | 0 | 0 |
| Dysgeusia | 2 (4) | 2 (4) | 0 | 0 | 0 | 0 | 0 | 0 | 0 | 0 | 0 | 0 |
| Paresthesia | 2 (4) | 2 (4) | 0 | 0 | 0 | 0 | 1 (2) | 0 | 1 (2) | 0 | 0 | 0 |
| Somnolence | 1 (2) | 0 | 0 | 1 (2) | 0 | 0 | 2 (4) | 0 | 0 | 2 (4) | 0 | 0 |
| Hypoesthesia | 0 | 0 | 0 | 0 | 0 | 0 | 2 (4) | 1 (2) | 0 | 0 | 1 (2) | 1 (2) |
| **Psychiatric disorders** | **12 (52)** | **8 (17)** | **2 (4)** | **3 (6)** | **2 (4)** | **0** | **1 (2)** | **1 (2)** | **1 (2)** | **0** | **0** | **0** |
| Euphoric mood | 3 (6) | 2 (4) | 1 (2) | 0 | 0 | 0 | 0 | 0 | 0 | 0 | 0 | 0 |
| Insomnia | 3 (6) | 2 (4) | 1 (2) | 0 | 0 | 0 | 0 | 0 | 0 | 0 | 0 | 0 |
| Abnormal dreams | 2 (4) | 2 (4) | 0 | 0 | 0 | 0 | 1 (2) | 1 (2) | 0 | 0 | 0 | 0 |
| Hallucination | 2 (4) | 1 (2) | 0 | 1 (2) | 1 (2) | 0 | 0 | 0 | 0 | 0 | 0 | 0 |
| **Gastrointestinal disorders** | **9 (19)** | **7 (15)** | **1 (2)** | **1 (2)** | **0** | **0** | **7 (15)** | **4 (9)** | **3 (6)** | **0** | **0** | **0** |
| Dry mouth | 2 (4) | 1 (2) | 1 (2) | 0 | 0 | 0 | 1 (2) | 1 (2) | 0 | 0 | 0 | 0 |
| Nausea | 1 (2) | 1 (2) | 0 | 0 | 0 | 0 | 3 (6) | 2 (4) | 1 (2) | 0 | 0 | 0 |
| Vomiting | 0 | 0 | 0 | 0 | 0 | 0 | 2 (4) | 2 (4) | 0 | 0 | 0 | 0 |
| **Infections and infestations** | **4 (8)** | **0** | **2 (4)** | **2 (4)** | **0** | **0** | **3 (6)** | **2 (4)** | **0** | **0** | **1 (2)** | **0** |
| Urinary tract infection | 2 (4) | 0 | 0 | 2 (4) | 0 | 0 | 1 (2) | 0 | 0 | 0 | 1 (2) | 0 |
| **Ear and labyrinth disorders** | **2 (4)** | **2 (4)** | **0** | **0** | **0** | **0** | **0** | **0** | **0** | **0** | **0** | **0** |
| Tinnitus | 2 (4) | 2 (4) | 0 | 0 | 0 | 0 | 0 | 0 | 0 | 0 | 0 | 0 |

AE = adverse event; MedDRA = Medical Dictionary for Regulatory Activities; TEAE = treatment-emergent AE.

^a^AE mapping was based on MedDRA version 20.1 thesaurus. Patients who experienced the same event more than once were counted once for the preferred term. Patients who experienced >1 event within a system organ class were counted only once in the system organ class.

^b^The number and percentage of patients in the system organ class represents all patients who had ≥1 AE (preferred term) in the system organ class. Only AEs that were reported in ≥2 patients in either treatment group in the nervous system, psychiatric system, and gastrointestinal system are displayed.
