# Supplementary material for: A Phase 2 Proof‐of‐Concept, Randomized, Placebo‐Controlled Trial of CX‐8998 in Essential Tremor
Source: Mov Disord. 2021 Mar 25;36(8):1944–9. doi: 10.1002/mds.28584 (PMC8451783; doi:10.1002/mds.28584)
Supplement: Supplementary file 5 — Table S4. TEAEs leading to discontinuation of study drug [file MDS-36-1944-s003.docx]

**Supplemental Table e-4.** TEAEs leading to discontinuation of study drug

| **Discontinuation^a^** | **CX-8998 (n=48)** | **Placebo (n=47)** |
| --- | --- | --- |
| Total patients with ≥1 event leading to discontinuation | **8 (17%)** | **2 (4%)** |
| Nervous system disorders | 4 (8%) | 1 (2%) |
| Dizziness | 2 (4%) | 0 |
| Depressed level of consciousness | 1 (2%) | 0 |
| Disturbance in attention | 1 (2%) | 1 (2%)^b^ |
| Head discomfort | 1 (2%) | 0 |
| Lethargy | 1 (2%) | 0 |
| Paresthesia | 1 (2%) | 1 (2%) |
| Psychiatric disorders | 4 (8%) | 0 |
| Insomnia | 2 (4%) | 0 |
| Abnormal dreams | 1 (2%) | 0 |
| Depression | 1 (2%)^b^ | 0 |
| Hallucination | 1 (2%) | 0 |
| Irritability | 1 (2%) | 0 |
| Suicidal ideation | 1 (2%)^b^ | 0 |
| General disorders and administration site conditions | 1 (2%) | 0 |
| Fatigue | 1 (2%) | 0 |
| Injury, poisoning, and procedural complications | 1 (2%) | 0 |
| Alcohol poisoning | 1 (2%)^b^ | 0 |
| Investigations | 1 (2%) | 0 |
| Electrocardiogram T wave abnormal | 1 (2%) | 0 |
| Metabolism and nutrition disorders | 1 (2%) | 0 |
| Hyperglycemia | 1 (2%) | 0 |
| Vascular disorders | 1 (2%) | 0 |
| Hypertension | 1 (2%)^b^ | 0 |
| Gastrointestinal disorders | 0 | 1 (2%) |
| Nausea | 0 | 1 (2%) |
| Vomiting | 0 | 1 (2%) |

AE = adverse event; MedDRA = Medical Dictionary for Regulatory Activities; TEAE = treatment-emergent adverse event.

^a^AE mapping was based on MedDRA version 20.0 thesaurus. Patients who experienced the same event more than once were counted once for the preferred term. Patients who experienced >1 event within a system organ class were counted only once in the system organ class.

^b^This event was considered unrelated to the study drug.
