# Supplementary material for: A Phase 2 Proof‐of‐Concept, Randomized, Placebo‐Controlled Trial of CX‐8998 in Essential Tremor
Source: Mov Disord. 2021 Mar 25;36(8):1944–9. doi: 10.1002/mds.28584 (PMC8451783; doi:10.1002/mds.28584)
Supplement: Supplementary file 6 — Table S5. TEAEs leading to dosage reductions [file MDS-36-1944-s004.docx]

**Supplemental Table e-5.** TEAEs leading to dosage reductions

| **Dosage Reduction^a^** | | | | | **CX-8998 (n=48)** | | | **Placebo (n=47)** | |
| --- | --- | --- | --- | --- | --- | --- | --- | --- | --- |
| Dosage reduced during study | | | | | 4 (8%) | | | 1 (2%) | |
| Due to AE | | | | | 3 (6%) | | | 1 (2%) | |
| Due to other reasons | | | | | 1 (2%)^b^ | | | 0 | |
| **Dosage Reductions Due to AEs** | | | | | | | | | |
| **Treatment Group** | **Patient Number** | **Dosage Reduction** | | | | **AE (MedDRA Preferred Term)^c^** | **Study Day of Last Dose** | | **Study Disposition** |
|  |  | **Study Day** | **From** | **To** | |  |  |  |  |
| CX-8998 | 26-001 | 25 | 10 mg BID | 8 mg BID | | Anxiety | 33 | | Completed study |
|  | 31-023 | 8 | 8 mg BID | 4 mg BID | | ECG T-wave abnormal | 8 | | Withdrawn (Day 8) Investigator decision |
|  | 33-004 | 8 | 4 mg BID | 2 mg BID | | Headache | 15 | | Withdrawn (Day 45) Lost to follow-up |
| Placebo | 38-018 | 15 | 4 capsules BID | 2 capsules BID | | Headache | 28 | | Completed study |

AE = adverse event; BID = twice daily; ECG = electrocardiogram; MedDRA = Medical Dictionary for Regulatory Activities; TEAE = treatment-emergent adverse event.

^a^Re-escalation of the dosage after dosage reduction was not allowed.

^b^Patient had inadequate drug supply at Visit 4.

^c^Events in all patients were considered related to the study drug.
